# InsideOut ReMind: A Sensory Device for Improving Memory Recall in Dementia

**DOI:** 10.1002/alz70861_108831

**Published:** 2025-12-23

**Authors:** Harshini I, Shivani M, Dalene Ephme Ben George, Allwyn S

**Affiliations:** ^1^ Sri Sivasubramaniya Nadar College Of Engineering, Chennai, Tamil Nadu India

## Abstract

**Background:**

Alzheimer’s disease severely impairs memory, cognition, and daily functioning, leaving patients confused, anxious, and socially isolated. Inspired by Pixar’s *Inside Out*, which personifies inner emotions, **InsideOut ReMind** brings personal memories to life through tactile interaction. This bedside device uses touch‐activated cubes to display familiar images and play comforting voice messages, reinforcing memory and reducing agitation. Family members can remotely personalize each cube with new photos and audio clips, ensuring an ongoing emotional connection.

**Method:**

The InsideOut ReMind system integrates an ESP32 Microcontroller with components such as capacitive touch sensors, 1.44″ TFT displays, DFPlayer Mini for audio, and WS2812B RGB LEDs for visual feedback. Each cube has a touch sensor that activates personalized images or text on the display and plays voice clips stored on a microSD card. The LED lights up in response to touch, enhancing the sensory experience. The system is powered by a Power Supply Module, and content is updated via a mobile app or PC. Family members can upload new photos and audio clips remotely. When a cube is touched, the ESP32 retrieves the image and voice clip, lights up the cube in a soothing color, and plays the message. The system features volume control and brightness adjustment. Content updates are made via Wi‐Fi, ensuring the system remains dynamic and relevant without opening the enclosure.

**Result:**

The InsideOut ReMind system helps Alzheimer’s patients by displaying personalized images and playing voice clips when the cubes are touched. This tactile interaction reinforces memory, helping patients recognize faces and recall relationships. The LED indicators further enhance the sensory experience. Though in development, the system aims to help patients with face recognition, memory retention, and emotional connection to loved ones

**Conclusion:**

The InsideOut ReMind system enhances dementia patients' lives by improving memory recall and fostering emotional connections. Providing a personalized, interactive experience, it empowers patients with autonomy, reduces caregiver dependency, and promotes social interaction

